# Factors associated with period of sick leave after gynecologic cancer treatment: a prospective cohort study

**DOI:** 10.1007/s00520-025-10116-5

**Published:** 2025-11-19

**Authors:** Yoshinori Tani, Keiichiro Nakamura, Hanako Sugihara, Shinsuke Shirakawa, Hirofumi Matsuoka, Naoyuki Ida, Junko Haraga, Chikako Ogawa, Eriko Eto, Shoji Nagao, Hisashi Masuyama

**Affiliations:** Department of Obstetrics and Gynecology, Dentistry and Pharmaceutical Sciences, Okayama University Graduate School of Medicine, 2-5-1 Shikata-Cho, Kita-Ku, Okayama, 700-8558 Japan

**Keywords:** Period of sick leave, Surgery plus chemotherapy, Six or more cycles of chemotherapy, Gynecologic cancer survivors

## Abstract

**Purpose:**

Gynecologic cancer is one of the most common malignancies in working-age women. This study aimed to investigate factors associated with period of sick leave after gynecologic cancer treatment in Japan.

**Methods:**

A prospective cohort study on period of sick leave was conducted among 207 cancer survivors who returned to work at the same workplace. Questionnaires were randomly distributed to patients aged under 65 years and more than one-year post-treatment. Clinical information was extracted from medical records, and the factors influencing the period of sick leave were analyzed using the Mann–Whitney U test and logistic regression analysis.

**Results:**

Surgery plus more than six courses of chemotherapy (number (*n*) = 41, 166.02 ± 146.84 days) led to a significantly longer period of sick leave than surgery without lymph node dissection (*n* = 64, 31.15 ± 30.47 days), surgery with lymph node dissection (*n* = 41, 55.56 ± 85.90 days), surgery plus less than six courses of chemotherapy (*n* = 21, 72.42 ± 56.07 days), and radiotherapy alone (*n* = 21, 58.85 ± 84.24 days) (OR: 2.63, 2.95, 2.67, and 2.08; 95% CI: 7.71–54.59, 18.17–92.94, 18.22–126.63, and 2.38–115.33; *p* = 0.009, *p* = 0.004, *p* = 0.009, and *p* = 0.041). gynecologic cancer survivors who experienced adverse effects after treatment had a significantly longer period of sick leave (OR: 8.50; CI: 52.98–84.98; *p* < 0.001). In univariate and multivariate analyses, patients who received surgery plus more than six courses of chemotherapy were most involved in long period of sick leave than other factors (OR: 11.20, and 16.997; CI: 4.86–25.08, and 5.51–52.35; *p* < 0.001, and *p* < 0.001).

**Conclusion:**

Patients with gynecologic cancer requiring long-term treatment required the most time to return to work.

**Supplementary Information:**

The online version contains supplementary material available at 10.1007/s00520-025-10116-5.

## Introduction

According to the data released by the Japanese National Cancer Center in 2019, the cumulative risk of cancer incidence for all people was 51.2% for women [1]. In 2020, the incidences of cervical cancer, endometrial cancer, and ovarian cancer with tubal and peritoneal cancer were 7,689, 13,113, and 8,004, respectively [2]. The most common age at diagnosis for cervical cancer was in the 40 s, and 50 s for endometrial cancer and ovarian cancer. The frequency of cervical cancer in less 70 s was 78.7%; endometrial cancer, 75.3%; and ovarian cancer, 75.0% [3]. The incidence of early stage (I and II) gynecological cancer is frequent, and the 5-year survival rates for patients with cervical cancer, endometrial cancer, and ovarian cancer were 92.3% and 76.2%, 93.9% and 87.6%, and 91.7% and 80.6%, respectively [4]. Gynecologic cancer patients with favorable prognosis and are associated with a large number of cancer survivors.

Treatment methods vary by cancer site. The Japan Society of Obstetrics and Gynecology has reported the frequency of cervical cancer treatment was surgery alone (31.3%), concurrent chemoradiotherapy (CCRT) (26.3%), radiotherapy (RT) alone (9.8%), surgery plus chemotherapy (surgery + Cx) (9.8%), and surgery plus CCRT (surgery + CCRT) (11.6%). The frequencies of the endometrial cancer treatments were surgery alone (58.5%), surgery + Cx (35.2%), surgery + RT (0.7%), Cx alone (1.6%), hormonal therapy alone (0.8%), and RT alone (1%). The frequency of ovarian cancer treatments was surgery + adjuvant Cx (49.2%), surgery alone (22.4%), Cx alone (2.8%), surgery, adjuvant Cx, and targeted therapy included neo-adjuvant Cx (14.3%). Surgery cases made up 52.7%, 94.6%, and 85.9% for cervical cancer, endometrial cancer, and ovarian cancer, respectively; and 21.4%, 36.1%, and 63.5% of patients with cervical cancer, endometrial cancer, and ovarian cancer, respectively, received additional treatment before and after surgery [3].

Cancer survivors face a variety of problems, one of which is returning to work. Return to work rates range from 42.9% to 95.2% among gynecologic cancer survivors in Japan [4]. At our institution, the return to work rates for gynecologic cancer survivors were 71.3% in 2015 and 82% in 2023. Compared to surveys conducted seven years ago, the frequency of returning to the same workplace increased [5, 6]. In a review of all cancer survivors, 62% returned to work after 12 months, and the average period of sick leave was 151 days [7]. Endo et al. reported that gynecologic cancer survivors returned to work after an average of 83 days of period of sick leave [8]. However, there have been no reports that have examined period of sick leave in detail. Therefore, we conducted a survey regarding period of sick leave and conducted research with the aim of providing a comfortable working environment not only for employees but also for employers.

## Methods

### Study population

Questionnaires were randomly distributed to all gynecologic cancer survivors aged < 65 years and ≥ 1 year post-treatment who visited Okayama University Hospital for consultation between October 5, 2023, and March 28, 2024. All participants were informed about the survey by their consulting doctors and provided written informed consent to participate in this study. All answers were voluntary. The completed questionnaires were collected in in-hospital collection boxes.

This study was approved by the Okayama University Ethics Committee (approval number: 2310–033) and conducted in accordance with the principles of the Declaration of Helsinki. A total of 282 gynecologic cancer survivors participated in the survey. Before treatment, 247 were employed and 35 were unemployed. Among them, 207 participants returned to the same workplace, while 40 either left or changed jobs. Patients who experienced relapse were excluded. Because the number of unemployed participants and those who changed jobs was relatively small, which could introduce statistical bias, the final analysis was restricted to the 207 patients who returned to the same workplace.

### Variables

Employment status at the time of diagnosis was divided into “self-employed,” “publicly employed,” “regularly employed” (permanent employment), and “non-regularly employed” (part-time, temporary, contract-based, and dispatched workers). We asked the participants to answer questions on employment patterns, working days per week, working hours per day, number of people in the workplace, personal income, household income, returning to the same workplace, and period of sick leave in an original questionnaire (supplementary Table 1). We also extracted data from medical records on age, marital status, presence of children, cancer site, cancer stage, cancer treatment, and adverse effects after treatment.

Treatment methods were examined in six groups: Surgery without lymph node dissection (LND) alone, Surg with LND alone, surgery + < 6 courses of Cx, surgery + ≧6 courses of Cx, surgery + RT (including CCRT), and RT alone (including CCRT). The average length of hospitalization or treatment was 9.4 days for surgery without LND alone, 11.5 days for surgery with LND alone, 90.4 days for surgery + < 6 courses of Cx, 172.8 days for surgery + ≧6 courses of Cx, 74.6 days for surgery + RT, and 57.3 days for RT alone. Period of sick leave was defined as the period from discharge from hospital to returned to the same workplace, and correlations between the period of sick leave and treatment methods, post-treatment side effects, and environmental factors were examined.

### Statistical analysis

Our study employed rigorous statistical analyses, including the Mann–Whitney U test for comparisons between groups and one-way ANOVA followed by Fisher’s protected least significant difference test for pairwise comparisons. Associations among the period of sick leave, treatment modalities, and post-treatment adverse effects in 207 gynecologic cancer survivors who returned to the same workplace were evaluated using the log-rank test. Univariate and multivariate analyses were performed using Cox proportional hazards models to identify factors associated with a period of sick leave exceeding 100 days. Statistical significance was set at *p* < 0.05. All analyses were conducted using SPSS software version 29.0 (IBM Corp., Armonk, NY, USA) to ensure accuracy and reliability.

## Results

**Fig. 1:**
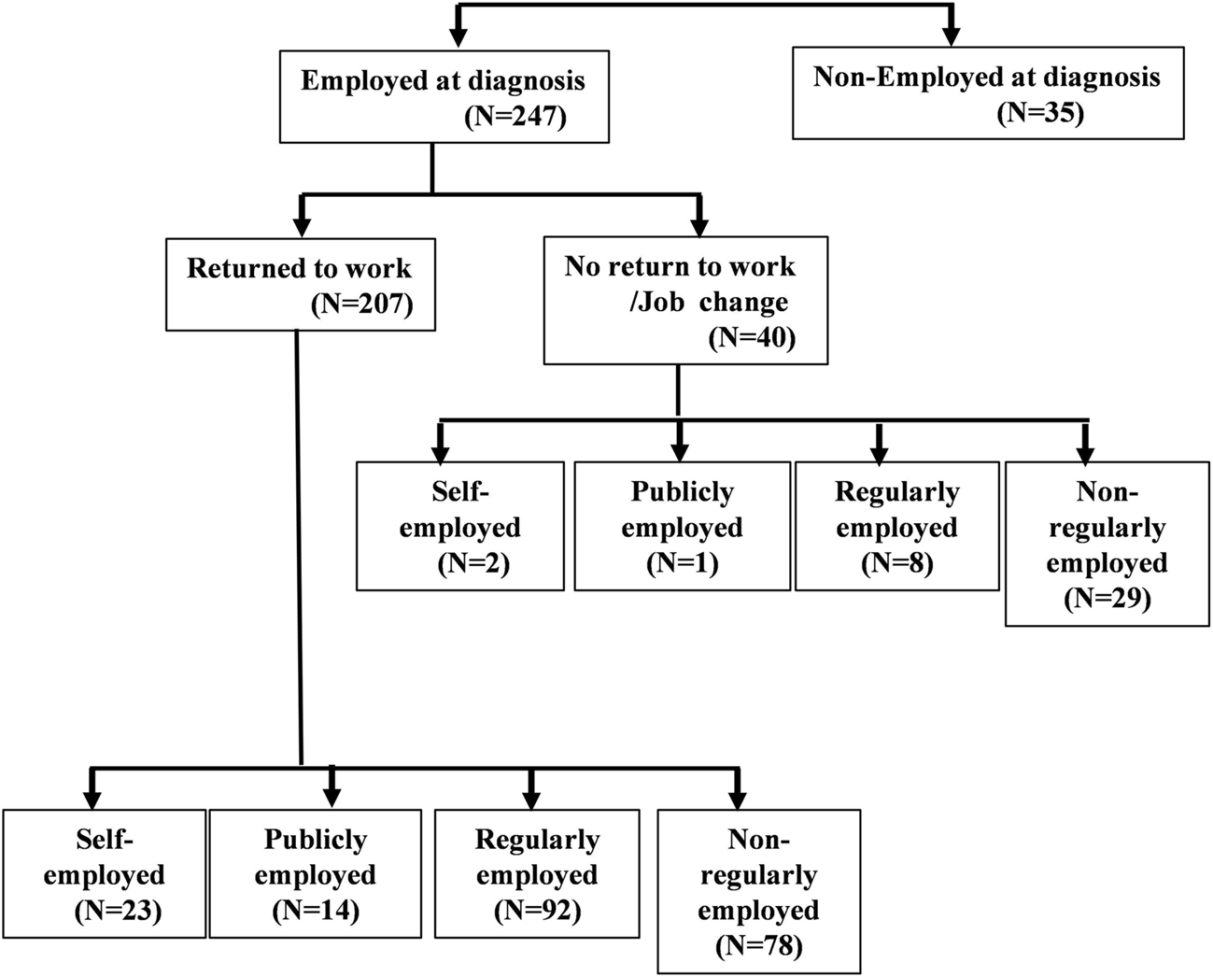
Employment status of cancer survivors who responded to the questionnaire at the time of diagnosis and after cancer treatment

The participants who returned to the same workplace, 23 (11.1%) were self-employed, 14 (6.8%) were publicly employed, 92 (44.4%) were regularly employed, and 78 (37.8%) were non-regularly employed. Among participants who quit or changed jobs, four were self-employed, three were publicly employed, nine were regularly employed, and 26 were non-regularly employed (Fig. 1).

**Fig. 2:**
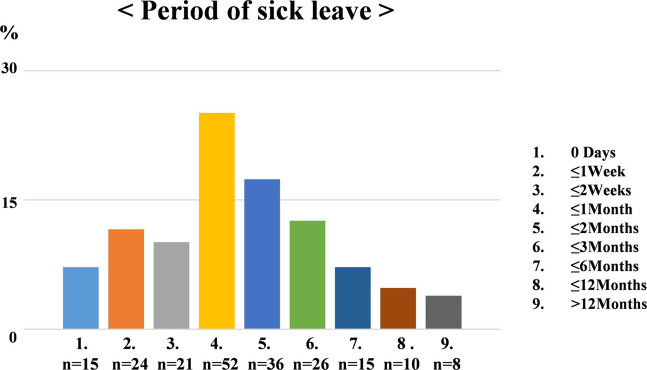
The period of sick leave after cancer treatment for all occupations

The mean age at the time of diagnosis was 47.7 years (median, 49 ± 8.3 years; range, 20–63 years), and the average number of years after treatment was 3.1. Table 1 details cancer survivors who returned to the same workplace. Participants with GC took an average of 76.3 days to return to the same job. The period of sick leave was 0 (7.2%), seven (11.6%), 14 (10.1%), 30 (25.1%), 60 (17.4%), 90 (12.6%), and 180 (7.2%) days for 15, 24, 21, 52, 36, 26, and 15 participants, respectively. Ten participants had less than 365 days (4.8%) and eight had more than 365 days (3.9%) of period of sick leave (Fig. 2).

**Table 1:** Patient characteristics

Age at diagnosis	Median, 47.7 Range; 20–63	
	Numbers	(%)
BMI		
< 18.5	22	10.6
18.5–24.9	121	58.5
25.0–29.9	35	16.9
30.0–34.9	21	10.1
35.0-	8	3.9
Marry		
Yes	165	79.7
No	42	20.3
Children		
Yes	140	67.6
No	67	32.4
Cancer site		
Cervical cancer	70	33.8
Endometrial cancer	91	44
Ovarian cancer	39	18.8
Other cancers	7	3.4
Stage		
Early	175	84.5
Advanced	32	15.5
Treatment		
Surgery (without LND)	64	30.9
Surgery (with LND)	41	19.8
Surgery + Chemotherpy (< 6 courses)	21	10.1
Surgery + Chemotherpy (≥ 6 courses)	41	19.8
Surgery + Radiation (included CCRT)	19	9.2
Radiation (included CCRT)	21	10.1
The work days per week		
≤ 3 days/week	11	5.3
4 days/week	22	10.6
5 days/week	154	74.3
≥ 6 day/week	20	9.7
Work time per day		
≤ 5 h/days	26	12.6
6–8 h/days	104	50.2
≥ 8 h/days	77	37.2
Workplace number of peoples		
≤ 5peoples	38	18.4
6–10 peoples	31	15
11–20 peoples	20	9.7
21–30 peoples	24	11.6
31–50 peoples	18	8.7
> 50 peoples	76	36.7
Person income (10,000yen)		
< 103	40	19.3
103–149	36	17.4
150–299	61	29.5
300–499	51	24.6
≥ 500	19	9.2
Household income (10,000yen)		
< 300	26	12.6
300–499	47	22.7
500–699	65	31.4
700–999	48	23.2
1,000–1,499	16	7.7
≥ 1,500	5	2.4

*BMI* body mass index, *LND* Lymph node dissection, *CCRT* concurrent chemoradiotherapy

**Fig. 3:**
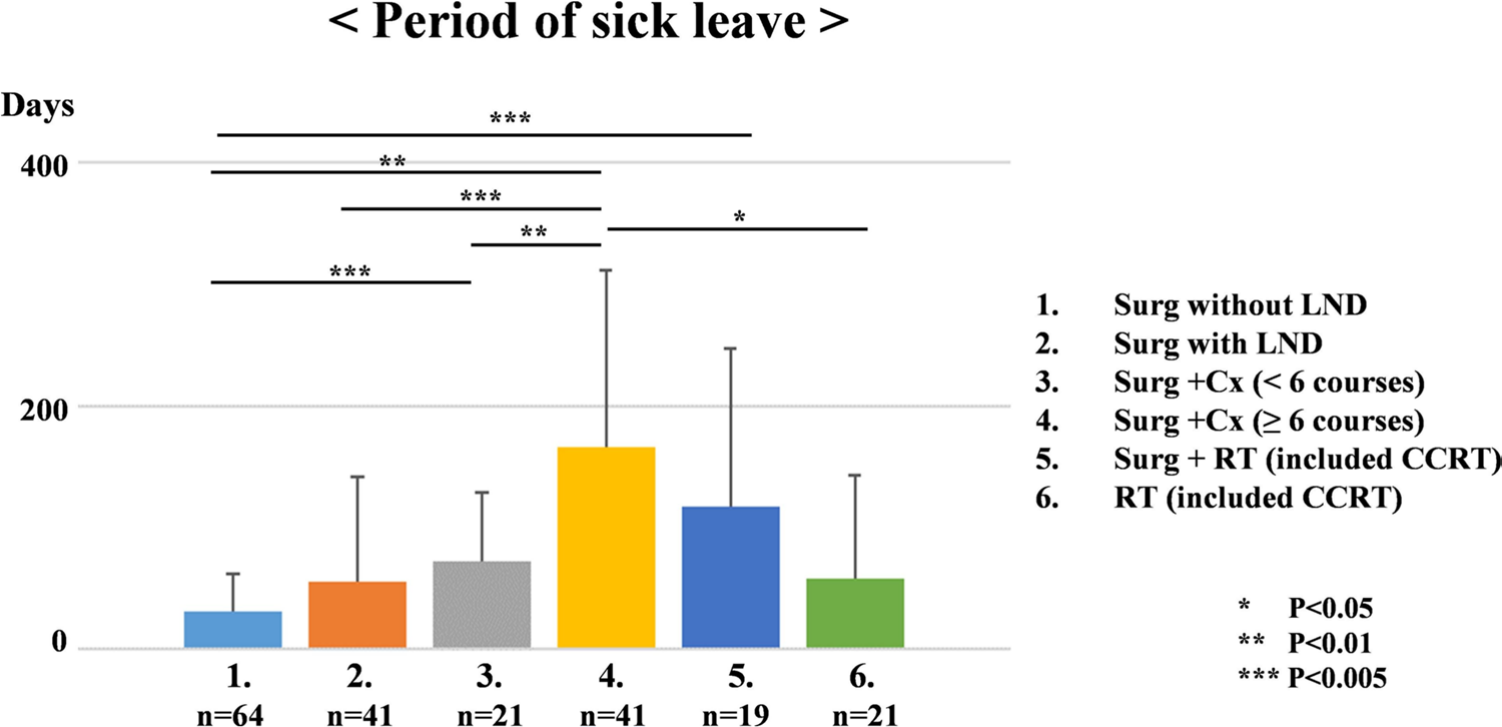
The period of sick leave after cancer treatment on treatment method [Surgery (Surg) without lymph node dissection (LND) alone, Surg with LND alone, Surg + < 6 courses of chemotherapy (Cx), Surg + > 6 courses of Cx, Surg + radiation (RT) (including concurrent chemoradiotherapy; CCRT), and RT alone (including CCRT)

The results showed that participants who received surgery + < 6 courses of Cx (72.42 ± 56.07 days: range, 0–180 days), surgery + ≧6 courses of Cx (166.02 ± 146.84 days: range, 0–400 days), and surgery + RT (117.68 ± 130.09 days: range, 0–400 days) had a significantly longer period of sick leave than participants who received surgery without LND alone (31.15 ± 30.47 days: range, 0–180 days) (odds ratio (OR): 8.67, 11.24, and 7.66; 95% confidence interval (CI): 55.82–89.02, 136.73–195.31; and 87.12–148.24; *p* < 0.001, *p* < 0.001, and *p* < 0.001). In particular, s surgery + ≧6 courses of Cx led to a significantly longer period of sick leave than surgery without LND alone, surgery with LND (55.56 ± 85.90 days: range, 0–400 days), surgery + < 6 courses of Cx, and radiotherapy alone (58.85 ± 84.24 days: range, 0–400 days) (OR: 2.63, 2.95, 2.67, and 2.08; CI: 7.71–54.59, 18.17–92.94, 18.22–126.63, and 2.38–115.33; *p* = 0.009, *p* = 0.004, *p* = 0.009, and *p* = 0.041). The group that received ≧6 courses of Cx took significantly longer to period of sick leave than the other treatment groups. We found that many gynecologic cancer survivors who received ≥ 6 courses of Cx treatment required nearly six months to period of sick leave (Fig. 3).

We investigated the surgical methods with and without LND in patients with gynecologic cancer survivors who underwent surgery alone. The group that underwent LND tended to period of sick leave later than the group that did not. However, we observed no relationship between the minimally invasive surgery group and time of period of sick leave (data not shown).

**Fig. 4:**
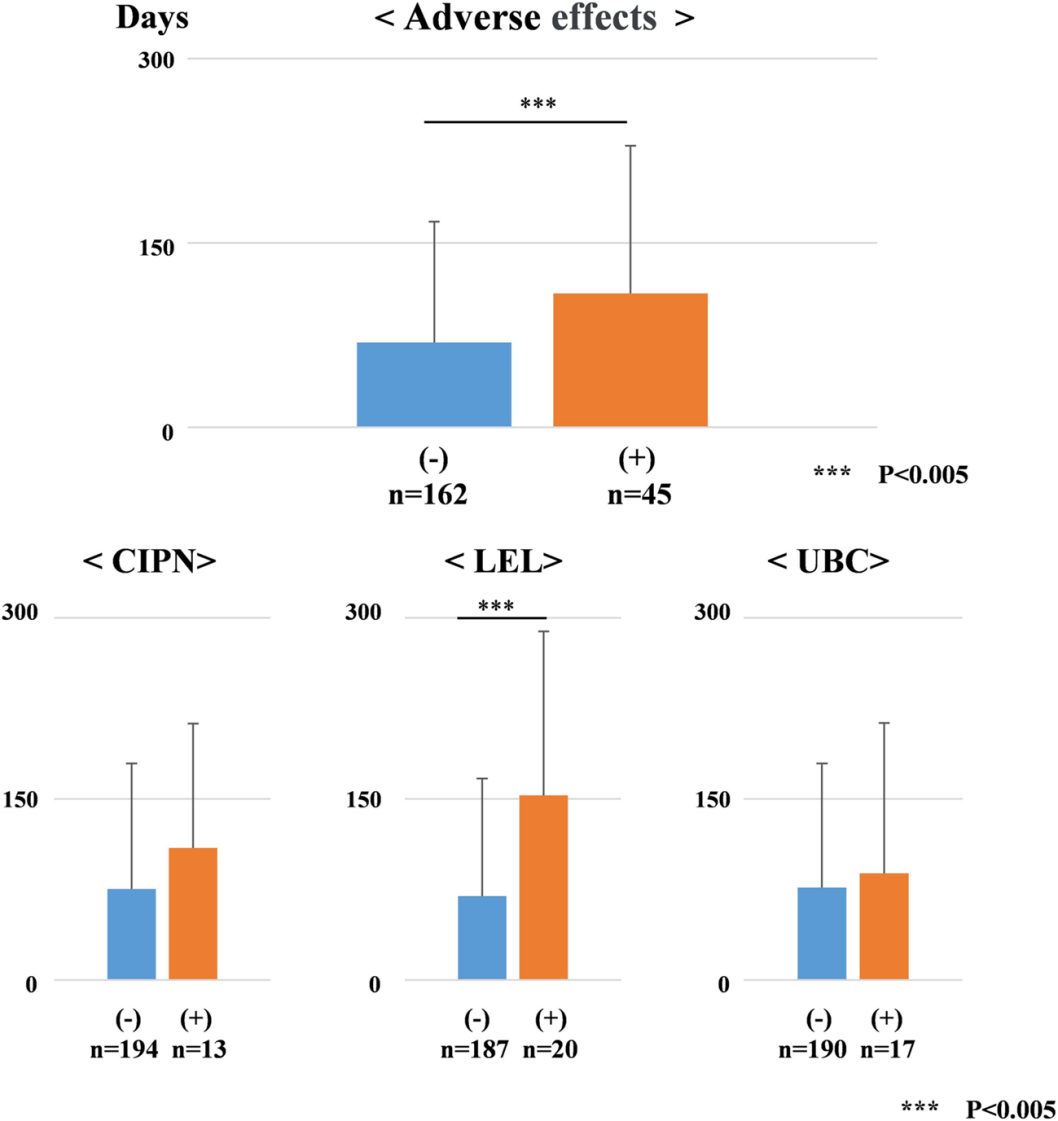
The period of sick leave after cancer treatment due to adverse effects after treatment. Current situation regarding return to work due to chemotherapy-induced peripheral neurotoxicity (CIPN), lower extremity lymphedema (LEL), and urological and bowel complications (UBC)

Treatment of gynecologic cancer often has adverse effects, including Cx-induced peripheral neurotoxicity (CIPN), lower extremity lymphedema (LEL), and urological and bowel complications (UBC). CIPN affects the lower and upper extremities with the use of certain chemotherapeutic agents, such as taxanes and platinum derivatives, and can cause loss of vibratory sensation and taste, paresthesia, weakness, tremors, and functional impairment [9–13].　LEL is also known to have a negative impact on patients’ quality of life, as it is associated with symptoms such as swelling, pain, numbness, and functional impairment [14–18]. We investigated the correlations between CIPN, LEL, UBC, and period of sick leave. After treatment, CIPN, LEL, and UBC were observed in 13, 20, and 17 participants, respectively. Five cancer survivors experienced multiple adverse events. Gynecologic cancer survivors who experienced adverse effects after treatment had a significantly associated with period of sick leave (OR: 8.50; CI: 52.98–84.98; *p* < 0.001). In particular, LEL was shown to be associated with period of sick leave (OR: 9.36; CI: 54.93–84.23; *p* < 0.001) (Fig. 4).

Univariate and multivariate analyses were used to analyze the factors that caused period of sick leave to last for more than 100 days to period of sick leave. In univariate analysis, advanced stage (OR: 3.643; CI: 1.548–8.570; *p* = 0.003), low personal income (OR: 2.5; CI: 1.094–5.712; *p* = 0.030), adverse effects after treatment (OR: 2.884; CI: 1.300–6.398; *p* = 0.009), and surgery + Cx (≧6 courses) were significantly associated with longer period of sick leave (OR: 11.209; CI: 4.863–25.809; *p* < 0.001). In multivariate analysis, short work time per day (OR: 0.141; CI: 0.022–0.902; *p* = 0.039), low personal income (OR: 4.317; CI: 1.213–15.362; *p* = 0.024), adverse effects after treatment (OR: 2.874; CI: 1.074–7.693; *p* = 0.036), and surgery + Cx (≧6 courses) (OR: 16.997; 95%CI: 5.518–52.352; *p* < 0.001) were significantly associated with longer PSL (Table 2). These results indicated that the longer the treatment period, the greater the effect of period of sick leave.

**Table 2:** Logistic regression analysis for more than 100 days to period of sick leave

Period	Odds ratio	Univariate analysis		Odds ratio	Multivariate analysis	
		95%Cl	*P*-value		95%Cl	*P*-value
Age (> 55 years)	1.851	0.824–4.161	0.136	1.185	0.422–3.323	0.747
BMI (≥ 30)	0.822	0.266–2.540	0.734	1.019	0.235–4.416	0.98
Marital status (None)	1.069	0.429–2.665	0.886	2.13	0.453–10.005	0.338
Children (None children)	1.054	0.478–2.323	0.897	0.854	0.212–3.445	0.825
Cancer site (Ovarian cancer)	0.949	0.363–2.486	0.916	0.334	0.093–1.196	0.092
Stage (Advanced stage)	3.643	1.548–8.570	0.003*	1.511	0.482–4.734	0.479
Employment pattern (Non regularly-employment)	1.468	0.692–3.114	0.317	1.284	0.458–3.598	0.635
The work days per week (≤ 3 days/week)	1.183	0.244–5.739	0.835	2.399	0.309–18.647	0.403
Work time per day (≤ 5 h/days)	0.657	0.185–2.327	0.515	0.141	0.022–0.902	0.039*
Workplace number of peoples (≤ Five peoples)	0.763	0.274–2.125	0.605	0.872	0.244–3.120	0.833
Person income (< 1,030,000 yen)	2.5	1.094–5.712	0.030*	4.317	1.213–15.362	0.024*
Household income (< 3,000,000 yen)	1.301	0.453–3.737	0.625	0.676	0.152–3.003	0.606
Adverse effect after treatment	2.884	1.300–6.398	0.009*	2.874	1.074–7.693	0.036*
Surgery plus chemotherapy (≥ 6 cycles)	11.209	4.868–25.809	< 0.001*	16.997	5.518–52.352	< 0.001*

*BMI* body mass index

*p < 0.05

## Discussion

Return to work rates vary widely by cancer site, with lung cancer and hematological malignancies having lower total return to work rates than other cancer sites [19, 20]. The return to work rates reported in 13 reviewed papers ranged from 53.8% to 95.2% in Japan [4]. It has been reported that Cx was negatively correlated with return to work, whereas minimally invasive surgery was positively correlated with return to work [21]. We reported that participants with cervical cancer treated with surgery + CCRT had a significantly higher rate of separation from employment than those treated with surgery alone on cervical cancer [17]. Although many studies have been conducted on return to work, there have been few reports on period of sick leave. In a review of cancer survivors, 62% returned to work after 12 months and the average period of sick leave was 151 days [7]. Endo et al. reported that patients with gynecologic cancer returned to work after an average of 83 days of period of sick leave [8]. Therefore, this study aimed to investigate the period of sick leave after gynecologic cancer treatment. We examined period of sick leave, treatment methods, and adverse effects after treatment in all occupations with gynecologic cancer survivors. The participants returned to the same workplace at an average of 77.6 days after treatment. In our analysis, the period of sick leave was similar to that reported by Endo et al. [8]. In the present study, no correlation was observed between period of sick leave and occupation with gynecologic cancer patients. Gynecologic cancer survivors who received surgery + Cx and surgery + RT had longer period of sick leave than those in the group that received surgery alone. In particular, the group that received more than six courses of Cx took significantly longer to period of sick leave than the other treatment groups. Cancer survivors who experienced adverse effects of LEL showed a significant relationship with period of sick leave. Interestingly, long-term treatment, it was shown to be more closely associated with period of sick leave than any other factor.

Our study has some limitations. The number of participants was relatively small, and the examinations were performed at a single facility. Further prospective studies involving more patients and facilities should provide definitive data to clarify the significance of our findings.

We found that long-term cancer treatment had the greatest impact on when the participants returned to work. period of sick leave is a major challenge for both employees and employers, and providing information in advance about it is an important element in building a good relationship.

## Conclusion

Gynecologic cancer patients who required long-term treatment experienced the longest period before returning to work. Therefore, establishing adequate social support systems is essential to facilitate a satisfactory return to work [5]. It is also important to communicate the expected duration of cancer treatment and the period of sick leave clearly to patients, and to provide them with appropriate information and guidance to help them smoothly reintegrate into their workplace and daily life after treatment.

## Supplementary Information

Below is the link to the electronic supplementary material.

ESM 1 Supplementary Material 1 (XLSX 13.6 KB)

## Data Availability

The datasets generated and analyzed during the current study are available from the corresponding author upon reasonable request.
